# Gut Microbiota: A New Strategy to Study the Mechanism of Electroacupuncture and Moxibustion in Treating Ulcerative Colitis

**DOI:** 10.1155/2019/9730176

**Published:** 2019-07-01

**Authors:** Daneng Wei, Lushuang Xie, Zhiqi Zhuang, Na Zhao, Biao Huang, Yong Tang, Shuguang Yu, Qizhi Zhou, Qiaofeng Wu

**Affiliations:** ^1^Acupuncture and Moxibustion College, Chengdu University of Traditional Chinese Medicine, Chengdu, Sichuan, 610075, China; ^2^Institute of Acupuncture and Homeostasis Regulation, Chengdu University of Traditional Chinese Medicine, Chengdu, Sichuan, 610075, China; ^3^Pharmacy College, Chengdu University of Traditional Chinese Medicine, Chengdu, Sichuan, 610075, China

## Abstract

Previous studies have confirmed that acupuncture and moxibustion is an effective way for treating ulcerative colitis (UC). However, the exact mechanism is unclear yet. In this study, DSS-induced UC mice were treated by electroacupuncture and moxibustion, and the genome of intestinal flora was subsequently detected by high-throughput sequencing in order to explore the detailed mechanism in terms of intestinal flora. The results indicated that the alpha diversity indices and beta diversity of intestinal flora were improved by electroacupuncture and moxibustion treatments, especially by the moxibustion treatment. These treatments inhibited* Streptococcus, Odoribacter, *and* Allobaculum *whereas it facilitated* Lactobacillus *on genus level. Further correlation analysis showed that the alpha diversity indices were positively correlated with the percentage of Treg cells in CD4^+^ cells but negatively correlated with the percentage of Th17 in CD4^+^ cells. These data indicated that both electroacupuncture and moxibustion can promote the intestinal flora diversity, providing a new view to understand the relationship between host and microbiome when using some external therapies.

## 1. Background

There are more than 100 trillion microbes inhabiting human gut, which are 10 times more than all of the cells in human body [1]. Coexistence between the host and gut microbiota is beneficial for shaping the mucosal and immune systems, protecting the intestinal epithelium from the harmful effects of pathogens, producing antimicrobial compounds, and then contributing to homeostasis [2, 3]. Besides, the diversity and composition of microbiota are dynamical. It can be altered by diet, environmental factors, exogenous probiotics, and antibiotic use [4–6]. Additionally, stress, lifestyle, and other behaviors would affect intestinal flora and gut homeostasis [6]. In recent years, an increasing number of gut microbiome has been detected with high-throughput sequencing. It is also found that gut ecosystem (dysbiosis) is frequently associated with increased susceptibility to infections as well as noncommunicable diseases like obesity, metabolic syndromes (e.g., diabetes and cardiovascular diseases), allergy, and other inflammatory diseases [7, 8]. Thus, regulating gut ecosystem becomes a significant target in adjusting intestinal microbial imbalance. Additionally, more evidence in recent studies also suggested that the mutual interaction between the intestinal flora and host has great impact on the body through the bidirectional communication pathway linking gut and microbiota with brain.

Ulcerative colitis (UC), usually invading colon and/or rectum, is one of the inflammatory bowel diseases (IBD). The predominant symptoms and characteristics of UC are abdominal pain, mucus stools, and bloody diarrhea. Currently, the incidence and prevalence rates of UC have not only increased obviously in Europe and North America [9], but also been rising rapidly in Asian countries [10, 11]. A long-term follow-up of UC in Hong Kong showed that the age-standardized incidence and prevalence rates of UC were 2.1/100,000 in 2006 and 26.5/100,000 in 2016, respectively. Notice that the rate has increased 12-fold in the past decade [12]. Therefore, UC has become a major global health problem that need to be solved urgently [13]. It is reported that UC can be induced by many pathogenesis including genetic susceptibility, immune dysfunction, unhealthy diet (excessive intake of carbohydrates and fat), infection, and environmental factors. While several recent studies suggested that imbalance of intestinal flora, including composition and abundance changes, closely correlated with the occurrence, progress, and outcome of UC [14, 15], which provided an opportunity to further understand the pathogenesis of UC, as well as some new treatment strategies.

Nowadays, the conventional medications for UC include aminosalicylates, steroids, antibiotics, and immunomodulators [13]. However, long-term medication cannot satisfy the patients' expectation of curing UC but decrease their compliance because of high recurrence rate and severe side effects, such as nausea, vomiting, headaches, and rash [13, 16]. Therefore, more and more patients resort to alternative and complementary medicine. As vital components of traditional Chinese medicine (TCM), acupuncture and moxibustion have attracted a great deal of interest, especially in China and several areas of Asia [17]. Evidence both from ancient medical books about TCM and modern studies showed that traditional external therapy treatments including acupuncture and moxibustion are able to improve the status of UC patients or models with few side effects [17–19]. In addition, a meta-analysis, collecting 11 randomized controlled trials or clinical controlled trials about acupuncture and moxibustion used for UC treatment in China, showed that acupuncture and moxibustion have a higher therapeutic efficacy than the control group using sulfasalazine (SASP) but less adverse reactions [20]. Animal experiments also showed that acupuncture and moxibustion therapy could ameliorate the symptoms of diarrhea, bloody stool, and weight loss and promote the rehabilitation of ulcer and the absorption of inflammation [21–23].

Lots of evidences confirmed that traditional acupuncture and moxibustion are effective on ameliorating UC symptoms and colonic inflammation. Our previous studies showed that both acupuncture and moxibustion could improve the disease active index (DAI, the evaluating method shown in Table 1) of DSS-induced colitis mice and affect the percentage of CD4^+^ T cells [24]. However, few studies involved intestinal flora of UC mouse which has higher homology than rat and pig [25]. T-helper 17 (Th17) and T-regulatory (Treg) cells (form Th17/Treg axis balance) are frequently found at barrier surfaces, particularly within the intestinal mucosa. They protect the host from pathogenic microorganisms and to restrain excessive effectors T-cell responses. Besides, the intestinal microbiome can also provide immunostimulatory signals activating innate and downstream adaptive immune responses, subsequently expanding Th17 cells and Tregs cells generation. Therefore, the aim of this study is to investigate (1) whether electroacupuncture and moxibustion can regulate the intestinal flora, as well as ameliorating UC symptoms or (2) whether the diversity of intestinal flora is related to Treg and Th17 cells. This study may provide new evidence from the view of ‘gut flora-host' to reveal the relationship of traditional external therapies and the gut flora, which can provide a new approach to view and explain the role of acupuncture and moxibustion in treating gastrointestinal diseases.

## 2. Results

### 2.1. The General Conditions of DSS-Induced UC Mice Were Improved after the Treatments of Electroacupuncture and Moxibustion

**Figure 1:**
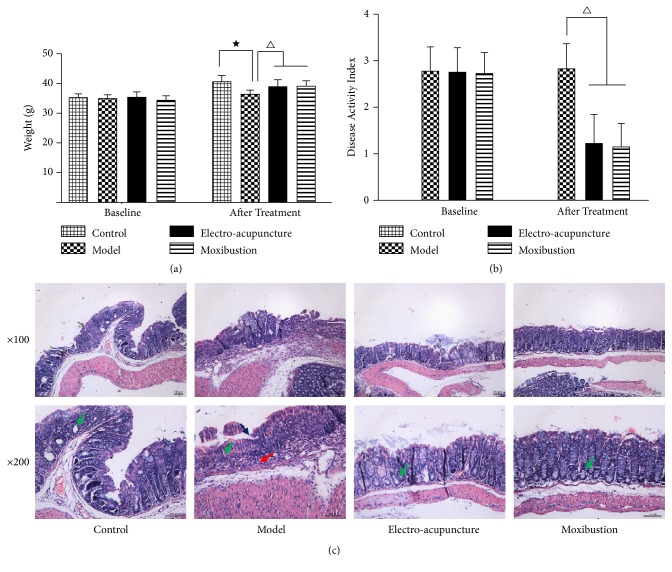
The therapeutic effects of electroacupuncture and moxibustion on DSS-induced mice. (a) is body weight before being administered with DSS (left) and after being treated with electroacupuncture and moxibustion (right) (n= 12/group); (b) is DAI score of the mice before being treated with electroacupuncture and moxibustion (left) and after being treated with electroacupuncture and moxibustion (right) (n= 12/group); (c) is H&E staining of colon from the four groups of mice (n=4~5/group). The presented values are the means ± SEM. ^★^*P*< 0.05 versus control, ^△^*P*< 0.05 versus model. Control represents healthy mice, model represents DSS-induced UC mice, electroacupuncture represents the mice induced by DSS and treated with electro-acupuncture, and moxibustion represents the mice induced by DSS and treated with moxibustion. Green arrows label glands, blue arrow labels mucous of model, and red arrow labels inflammatory cell.

The body weight of each experimental mouse was about the same (Figure 1(a)). After administration of the 3% DSS solution, the DSS-induced UC model mice showed a significant weight loss (Figure 1(a)), loose even watery excrement and with an increased DAI score (Table 2 and Figure 1(b)). After treating with electroacupuncture and moxibustion, the general conditions of UC mice were improved (Table 2). The body weight of the mice in electroacupuncture and moxibustion group was significantly increased compared to that of the mice in the model group (P<0.05) (Figure 1(a)), while the DAI score was decreased after interventions compared with UC group (P<0.05) ((Table 2 and Figure 1(b)). However, there was no significant difference between the electroacupuncture group and moxibustion group in terms of DAI scores (P> 0.05) ((Table 2 and Figure 1(b)). In view of colonic morphology, the mucosa of UC mice was obviously edematous and congestive. The structure of glands in mucosa was destroyed. Mucosal and submucosal layers were incrassated and infiltrated with a large number of inflammatory cells. However, the colonic morphology changes of the UC mice were significantly improved after the electroacupuncture and moxibustion treatments. Meanwhile, structures of glands and mucosal epitheliums obviously recovered. But no significant difference was observed between the electroacupuncture group and moxibustion group (Figure 1(c)).

### 2.2. The Intestinal Microbial Diversity of UC Mice Was Improved by Electroacupuncture and Moxibustion Treatments

#### 2.2.1. Species Annotation and Evaluation

**Figure 2:**
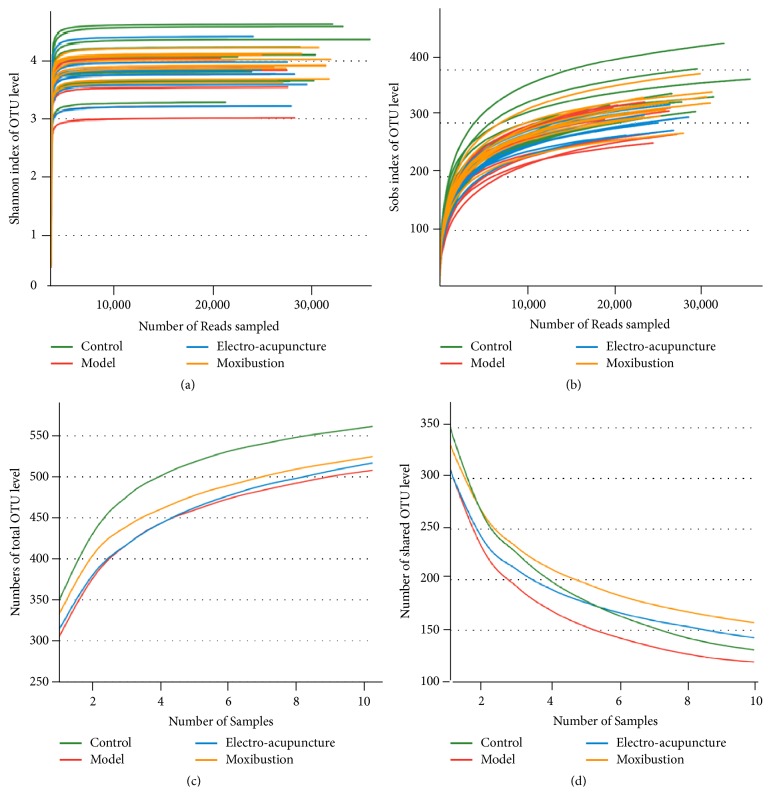
Evaluation of sequenced data from fecal samples. (a) is rarefaction curves calculated from the flora sequenced data using Shannon index of OTU level; (b) is rarefaction curves calculated from the flora sequenced data using Sobs index of OTU level; (c) is Pan species of intestinal flora in each group on the OTU level; (d) is core species of intestinal flora in each group on the OTU level. The number of samples is 10 in each group.

**Figure 3:**
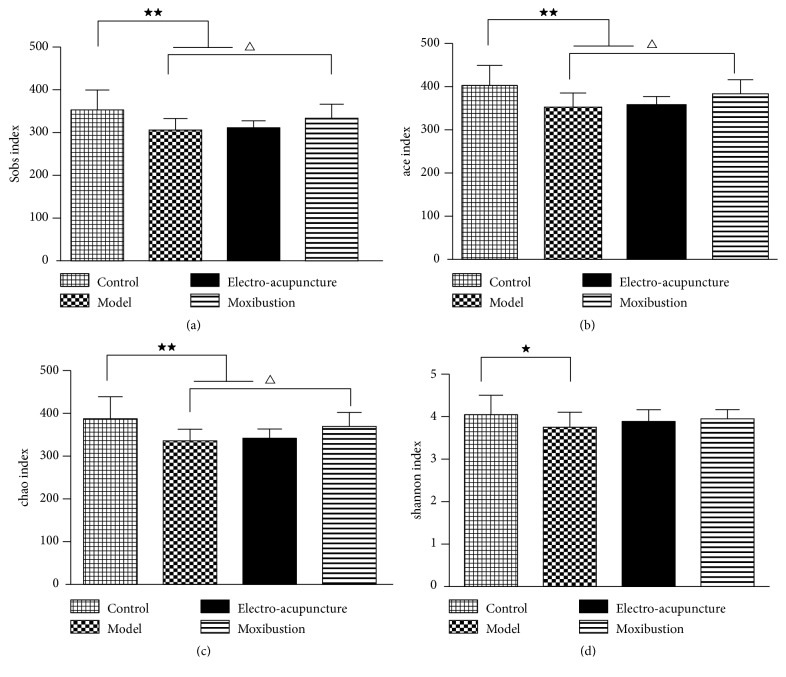
The effects of electroacupuncture and moxibustion on *α* diversity index. (a), (b), and (c) are community richness index of intestinal flora on OTU level (Sobs, Ace, and Chao index, respectively); (d) is community diversity index of intestinal flora on OTU level (Shannon index). All analysis are calculated on OTU level and the presented values are the means ± SEM. ^★^*P*< 0.05 versus control, ^★★^*P*< 0.01 versus control, and ^△^*P*< 0.05 versus model.

The number of observed species increased with the amount of sequencing data rising in the rarefaction curve. When the amount of sequencing data reached a certain value, the rarefaction curve would show a flatly change. It indicated that the diversity index slowly increased while the platform had been achieved (Figures 2(a) and 2(b)). The Pan/Core species analysis showed that the abundance of total species and core species in UC model mice decreased, but it was improved by electroacupuncture and moxibustion treatments. Among which, the amount of species in the moxibustion group was increased more than that of the electroacupuncture group (Figures 2(c) and 2(d)). Compared with the control group, the Ace, Chao, and Sobs indices of the intestinal flora in the model, electroacupuncture, and moxibustion groups reduced in terms of alpha diversity analysis, especially the model and electroacupuncture groups (P<0.01). After electroacupuncture and moxibustion treatments, the alpha diversity indices of both electroacupuncture and moxibustion groups were increased, especially after the moxibustion treatment (P<0.05) (Figures 3(a), 3(b), and 3(c)). Besides, the Shannon index, which reflects community diversity of intestinal flora, showed a significant decrease in UC model (P<0.05). However, there were no differences among the model, electroacupuncture, and moxibustion groups according to Shannon index (Figure 3(d)).

#### 2.2.2. Sample Comparative Analysis of Intestinal Flora

**Figure 4:**
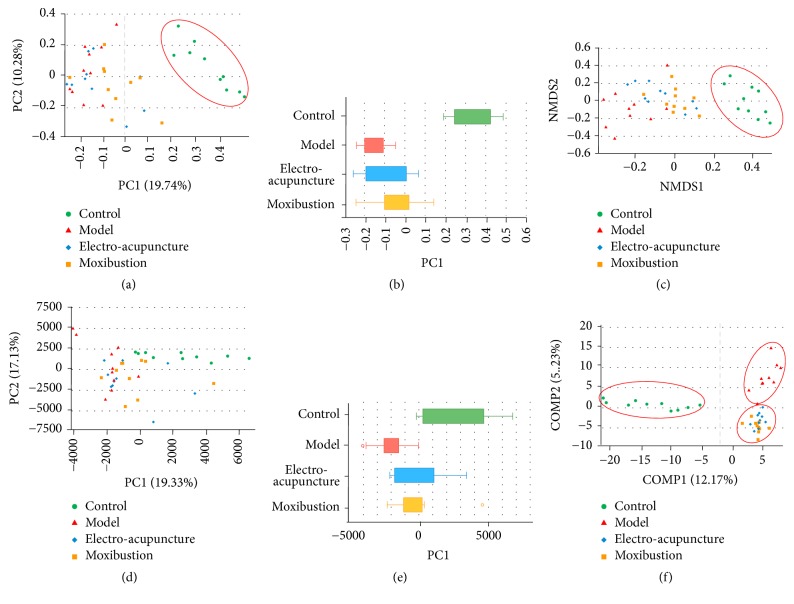
The effects of electroacupuncture and moxibustion on *β* diversity of intestinal flora. (a) and (b) are PCoA analysis of intestinal flora; (c) is NMDS analysis of intestinal flora; (d) and (e) are PCA analysis of intestinal flora. (f) is grouping comparison of intestinal flora using partial least squares discriminant analysis (PLSDA). The distance between the different colored samples represents the similarity of microbiota composition in different groups, and a closer distance indicates higher similarity. All analysis was conducted on OTU level and the number of samples is 10 in each group.

Beta diversity analysis mainly includes the PCoA, NMDS, and PCA analysis, which is used to analyze the distributions of intestinal flora. The results of beta diversity analysis showed that the intestinal flora in different groups showed some differentiation. Within the figures of beta diversity analysis, the distances among the different samples represent the similarity of the composition and abundance of intestinal flora. A closer distance indicates higher similarity. The results of the beta diversity analysis showed that the model group was significantly different from the control group (Figures 4(a), 4(c), and 4(d)). The samples of both the electroacupuncture group and moxibustion group were distributed in the middle of the control group and UC group. Although there were overlaps among the electroacupuncture group, moxibustion group, and the model group in distributions (Figures 4(a), 4(c), and 4(d)), the PCoA and PCA results showed that the two intervention groups seemed to be much closer to that of the control group (Figures 4(b) and 4(e)). Additionally, the distance between the control group and moxibustion group was the closest (Figure 4(b)), suggesting higher similarity between them. Further grouping analysis was carried out by partial least squares discriminant analysis (PLSDA). The results showed that the sample distributions of the model, electroacupuncture, and moxibustion groups were significantly different from those of the control group. What is more, these three groups differed from each other too (Figure 4(f)).

#### 2.2.3. Community Composition Analysis

**Figure 5:**
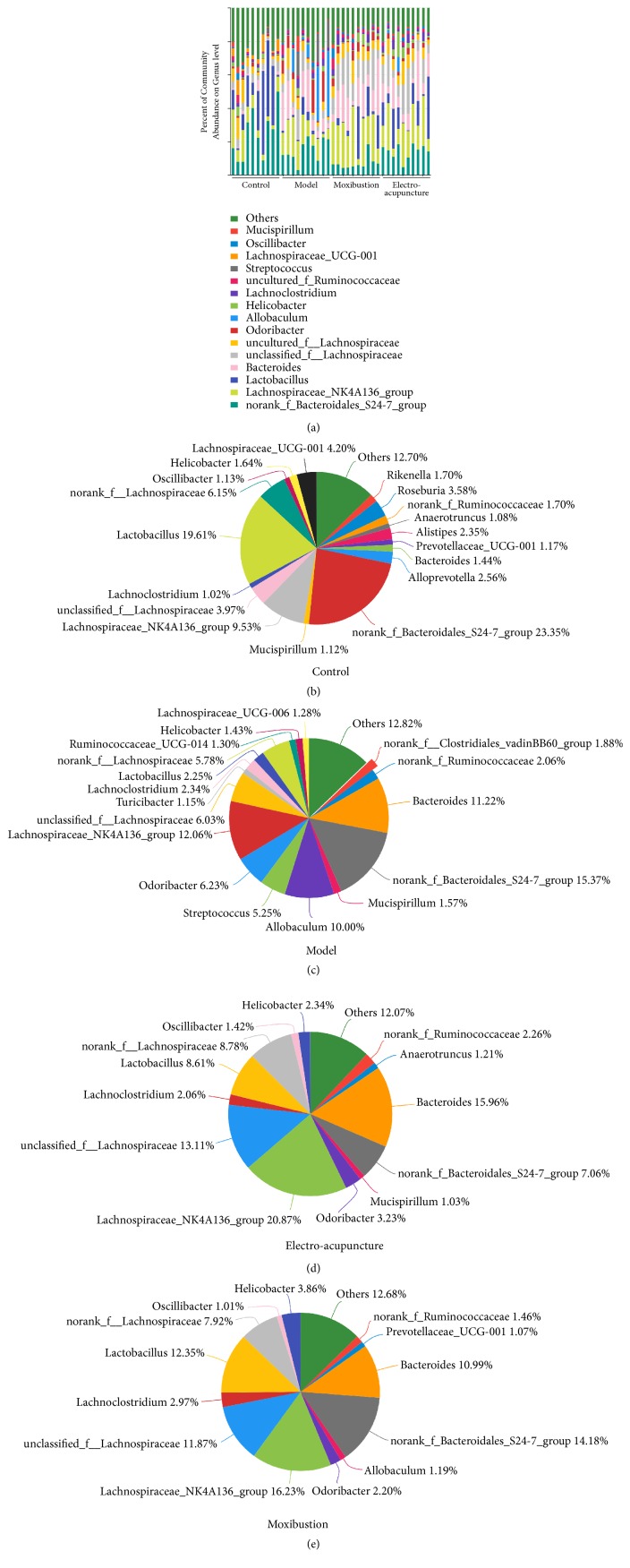
The effects of electroacupuncture and moxibustion on composition of intestinal flora. (a) is the community components of intestinal flora. (b), (c), (d), and (e) are community components of intestinal flora in different groups (control, model, electroacupuncture, and moxibustion, respectively). The number of samples was 10 in each group and all of these are analyzed on genus level.

**Figure 6:**
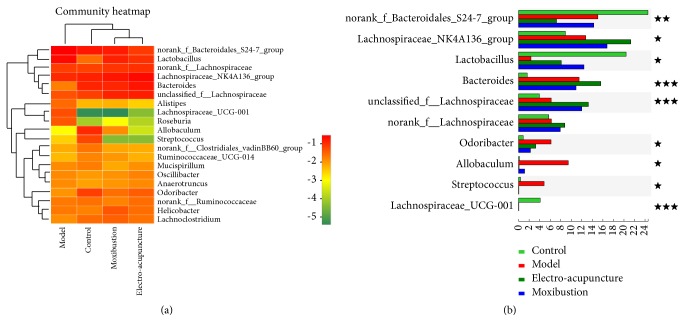
Heat map and community comparison of intestinal flora. (a) is heat map. The right is the name of communities and the left and upper are the community clustering tree and the sample clustering tree, respectively. The color depth represents different community abundance in heat map. (b) The difference analysis of high abundance communities among groups on genus level. ^★^ represents the* P* value of inter-group difference is less than 0.05, ^★★^represents the* P* value of inter-group difference is less than 0.01, and ^★★★^ represents the* P* value of inter-group difference is less than 0.001. The number of samples is 10 in each group.

These high abundance communities of flora were analyzed on genus level and presented with forms of bar plot, pie plot, and heatmap. The results showed that the* Lachnospiraceae_UCG-001* in the control group accounted for 4.20%, but few in the other three groups (Figures 5(a), 5(b), and 6(a)), while the* Streptococcus* (accounting for 5.25%) was the highest in the model group (Figures 5(a), 5(c), and 6(a)). There were some other communities increasing significantly in the model group, including* Bacteroides* (11.22%),* Odoribacter* (6.23%), and* Allobaculum* (10.00%), but the* Lactobacillus*, accounting for 2.25%, was significantly less than other groups (Figures 5(a), 5(c), and 6(a)). Both the two interventions could improve the flora imbalance. The* Lactobacillus *was increased via electroacupuncture and moxibustion treatment and accounted for 8.61% and 12.35%, respectively, but the* Odoribacter* and* Allobaculum* were decreased (Figures 5(a), 5(d), 5(e), and 6(a)). The* norank_f_Bacteroidales_S24-7_group* of the model, electroacupuncture, and moxibustion groups were less than that of the control group, while the* Lachnospiraceae* proportion of the electroacupuncture and moxibustion groups was more than that of the model group (Figures 5(a), 5(b), 5(c), 5(d), 5(e), and 6(a), Table 3).

#### 2.2.4. Species Difference Analysis

Clustering analysis in heat map was carried out to compare the composition and abundance difference of intestinal flora in different groups. The result showed that the electroacupuncture and moxibustion groups were more similar to the control group (Figure 6(a)). Furthermore, the inter-group difference of flora communities with high abundance was analyzed on genus level. The abundance of* Lachnospiraceae_NK4A136_group*,* norank_f_Bacteroidales_S24-7_group*,* Lactobacillus*,* Bacteroides*,* unclassified_f_Lachnospiraceae*,* Odoribacter, Allobaculum, Streptococcus*, and* Lachnospiraceae_UCG-001* in different groups showed significant inter-group difference (P<0.05 or P<0.01 or P<0.001) (Figure 6(b)). In detail, the abundances of* Bacteroides*,* Odoribacter, Allobaculum, and Streptococcus* in UC mice were all obviously increased (P<0.05 or P<0.01), but those* of norank_f_Bacteroidales_S24-7_group*,* Lactobacillus*, and* Lachnospiraceae_UCG-001 *in UC mice were all obviously decreased (P<0.05 or P<0.01). After the electroacupuncture and moxibustion treatment, some of these flora abundance changes were improved. Compared with the model group, the* Odoribacter, Allobaculum, and Streptococcus* of the electroacupuncture and moxibustion groups were all obviously decreased (P<0.05), but the* Lactobacillus, Lachnospiraceae_NK4A136_group, *and unclassified* _f_Lachnospirac-eae *of the treatment groups were all increased, especially the* Lactobacillus *in the moxibustion group and* Lachnospiraceae *(including* Lachnospiraceae_NK4A136_group *and unclassified_*f_Lachnospiraceae) *in the electroacupuncture group (P<0.05 or P<0.01).

### 2.3. Correlation Analysis between Treg/Th17 Cells and the Diversity of Intestinal Flora

**Figure 7:**
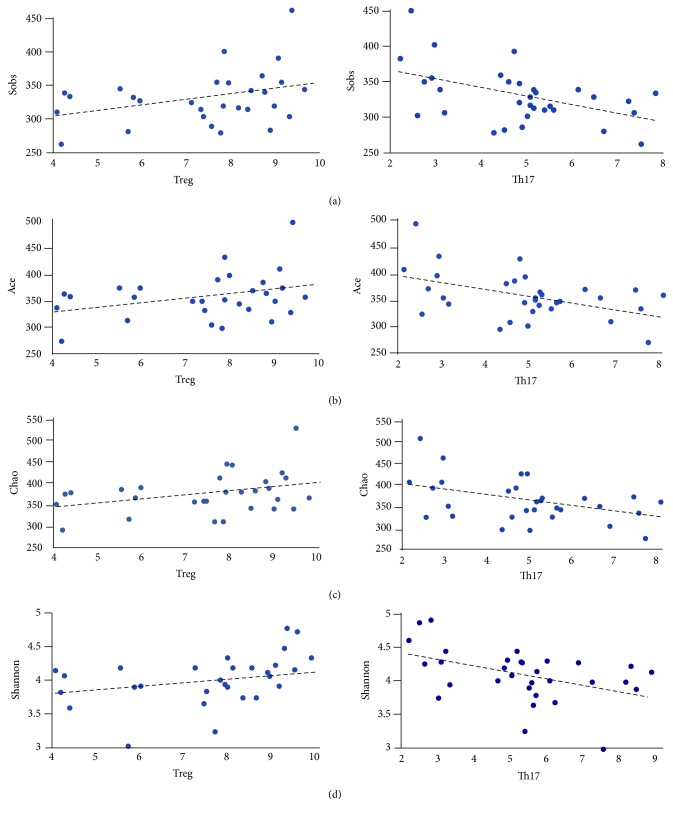
Scatter diagram of correlation between intestinal flora diversity and Treg/Th17. These spots represent cross-points between different intestinal flora diversity index and levels of Treg/Th17. The abscissa represents Treg/Th17 results tested by flow cytometry and the ordinate represents gut flora diversity. (a) is correlation between Sobs index and Treg/Th17 cell. (b) is correlation between Ace index and Treg/Th17 cell. (c) is correlation between Chao index and Treg/Th17 cell. (d) is correlation between Shannon index and Treg/Th17 cell.

A bivariate correlation (mainly Pearson correlation) was used to analyze the correlation between Treg and Th17 cells (original results of the percentage of T cells and Th17 cells in CD4+ are shown in supplementary material (available here)), which are two main subtypes of CD4^+^ T cells associated with the occurrence and progress of UC in previous studies, and the diversity of intestinal flora. The results revealed that the Treg cells were positively correlated with Sobs, Ace, and Chao index (P<0.05), but the level of Th17 was negatively correlated with Sobs, Ace, and Chao index (P<0.01) (Figures 7(a), 7(b), and 7(c)). Besides, the Th17 cells were negatively correlated with Shannon index (P<0.05), but the correlation analysis between the Treg cells and Shannon index was no statistical difference (Figure 7(d)). These results indicated that the flora diversity increased along with the Treg cells while it decreased along with Th17 cells. According to the alpha diversity analysis, indices (including Sobs, Ace, Chao, and Shannon) decreased in model group but were then obviously improved by the electroacupuncture and moxibustion.

## 3. Discussion

### 3.1. Characteristics of Intestinal Flora in UC

Intestinal microbiota plays an essential role in the physiology, nutrition, and immunity of human hosts. Actually, that the intestinal microbiota maintains relative stability contributes to the inner homeostasis of gut [26]. Increasing studies indicated that dysregulation of the intestinal flora is closely associated with pathogenesis of diabetes, obesity, hyperlipidemia, cardiovascular disease, colon cancer, IBD, IBS, and other intestinal diseases. Lots of evidence has confirmed that variations of microbial composition and abundance exist in IBD patients or experimental models compared with healthy controls [27]. The fecal microbial genes abundance of IBD patients including UC and Crohn's disease (CD) is 25% less than the healthy individuals and the microbial compositions also have a distinct difference [28]. Although CD and UC both belong to IBD, the mucosal microbiota of UC is also different from that of CD. For instance, both the* Faecalibacterium prausnitzii* increasing and flora diversity reducing are observed in CD but not in UC [29]. Additionally, the mucosa-associated intestinal flora of ulcerated regions was significantly different from that of nonulcerated regions in UC, especially the* lactobacilli* and the* Clostridium leptum *subgroup, which were reported to cause UC [30]. Although the composition of human's commensal intestinal microbiota differed from the mice, the destruction of intestinal microbial diversity involved in the abundance and composition of the flora, is similar in gut inflammation diseases. After being colonized by the gut microbiota isolated from UC patients, germ-free interleukin 10-deficient mice emerged obvious colitis symptoms with a proinflammatory gene expression and a decrease in microbial diversity [31]. The* Klebsiella pneumoniae* and* Proteus mirabilis* were also detected in T-bet(-/-)×Rag2(-/-) ulcerative colitis (TRUC) mice, and these TRUC-derived communities could trigger colitis in both Rag2(-/-) and WT mice[32]. Moreover,* Fusobacterium varium* isolated from UC patients could kill Vero cells, and its culture supernatants caused UC-like lesions in mice [33].Various Streptococcus mutants with a high detection rate in UC patients were also confirmed to aggravate the colitis [34]. In summary, the decrease of flora diversity and changes of community composition and abundance are closely associated with the pathogenesis of UC.

In this study, the alpha diversity indices (Ace, Chao, Sobs, and Shannon) of intestinal flora in UC mice were significantly decreased. Meanwhile, the beta diversity analysis (PCA, PCoA and NMDS) also showed that intestinal flora distribution of UC mice was different from control mice. These results indicated that the diversity of intestinal flora was broken in UC. Furthermore, the flora community composition of UC mice was changed as well. The* Bacteroides*,* Odoribacter, Allobaculum, and Streptococcus* of UC mice were all obviously increased, while the* norank_f_Bacteroidales_S24-7_group*,* Lactobacillus*, and* Lachnospiraceae_UCG-001* were all obviously decreased. These results were consistent with other studies. For instance,* Lactobacillus*,* Bacteroidales*,* Lachnospiraceae,* and* Streptococcus* have been confirmed to relieve or promote UC [34–36] and high abundance of* Odoribacter* was detected in the colonic tumor-bearing mice [37]. However, the correlation between* Allobaculum *and UC remained unclear.

### 3.2. Electroacupuncture and Moxibustion Ameliorates DSS-Induced Colitis

Currently, there are very few specific treatments for UC but some symptomatic treatments, and they may cause other untoward effect. Therefore, hunting some effective and safe treatments for UC has been urgent and imperative. Because of superior availability and relatively low clinical side-effects, acupuncture and moxibustion have become important complementary and alternative therapies for UC. There is a meta-analysis showing that acupuncture and moxibustion were effective on symptom control of IBD (including UC) [18]. Another meta-analysis including 5 randomized clinical trials (RCTs) also proved that moxibustion generated more favorable effects than conventional drug therapies [19]. Besides, both clinical and animal studies found that acupuncture and moxibustion can significantly relieve symptoms of UC and decrease DAI scores. For instance, Stefanie Joos et al. reported that the colitis activity index (CAI, same as DAI) decreased from 8.0 to 4.2 in acupuncture group but only 2 (from 6.5 to 4.8) in sham group [38]. Our previous studies showed that both electroacupuncture and moxibustion can improve the intestinal pathological morphology of the DSS-induced UC model [24]. The levels of IL-2, IL-6, IL-10, IL-17A, IL-17F, and TGF-*β* in plasma were also improved by electroacupuncture and moxibustion [24]. In this study, we found that electroacupuncture and moxibustion can increase the body weight, decrease the DAI scores, and promote the recovery of colonic mucosa. These results reconfirmed the curative effects of electroacupuncture and moxibustion on UC.

### 3.3. Restoration of Intestinal Flora in UC By Electroacupuncture and Moxibustion

The impairment of intestinal flora may result in the damage of enteric mucosa and various inflammations, so restoring the homeostasis of intestinal flora is vital for UC treatment. The fact that increasing intestinal flora diversity via fecal microbiota transplantation (FMT) is beneficial for IBD patients has been approved worldwide [39]. FMT could increase phylotype richness of intestinal flora and shift UC flora communities, characterized by increase of* Enterobacteriaceae* and decrease of* Bacteroidetes*,* Firmicutes*, and* Verrucomicrobia *[39]. Furthermore, supplementing the mixture of* Lactobacilli* and* Bifidobacteria* is helpful to reduce DAI scores of relapsing UC patients and improve rectal bleeding [40]. Prior administration of* Lactobacillus* and* Bifidobacterium* is also effective for relieving mucosal inflammation and modulating the fecal anaerobic bacteria in DSS-induced colitis mice [41]. Thus, restoring the diversity of intestinal flora and rebalancing microbial homeostasis have become a new strategy for UC treatment.

Although lots of clinical trials and animal experiments showed that acupuncture and moxibustion are beneficial in relieving the symptoms and signs of UC, few studies were designed to explore if this therapeutic effect is related to restoring the homeostasis of intestinal flora. Our previous study showed that electroacupuncture could increase the intestinal flora abundance and diversity of UC rats, promote the* Lactobacillus sp. *and* Lachnospiraceae bacterium, *and inhibit the* Clostridium bifermentans *[42]. However, we only roughly observed the changes of intestinal flora due to the limitation of denaturing gradient gel electrophoresis (DGGE). Additionally, Qin Qi et al. found that moxibustion therapy could modulate the gut microbiome of UC rats and alleviate the colonic inflammation [43]. In order to exhaustively understand the effects of electroacupuncture and moxibustion on intestinal flora as well the difference between them, we designed this study and chose the Illumina-MiSeq sequencing to detect and identify whole genome of intestinal flora even with a low abundance.

In this study, the results showed that the diversity of intestinal flora of UC mice, as well as compositions, was restored by electroacupuncture and moxibustion. Both of electroacupuncture and moxibustion could improve the alpha diversity indices and beta diversity distributions of UC mice. This effect on intestinal flora was similar to FMT for IBD patient [39]. Meanwhile, both electroacupuncture and moxibustion inhibited* Odoribacter, Allobaculum, *and* Streptococcus *but facilitated* Lactobacillus* and* Lachnospiraceae *(including* Lachnospiraceae_NK4A136_group and unclassified_f_Lachnospiraceae)*. Furthermore,* Lactobacillus* of the moxibustion group and* Lachnospiraceae *of the electroacupuncture group were increased obviously, but* Bacteroides* of them was almost unchanged. These results suggested that* Odoribacter, Allobaculum *and* Streptococcus*,* Lactobacillus, *and* Lachnospiraceae* played vital roles in restoring intestinal flora homeostasis of UC mice through electroacupuncture and moxibustion therapies. To some extent, the effects of these traditional Chinese medical methods on compositions and abundances of flora were similar to supplementing probiotics mainly containing* Lactobacilli* and* Bifidobacteria* [40, 41] and FMT [39] for UC patients or models. Thus, restoring the diversity of intestinal flora and rebalancing the major communities (inhibiting pathogens and promoting probiotics) may be the potential mechanism for electroacupuncture and moxibustion to relieve symptoms and signs of UC. Recently, Qin Qi et al. found that rats treated with moxibustion and mesalazine had significantly lower levels of the dominant phyla* Proteobacteria* and the genera* Saccharibacteria*,* Sphingomonas*, and* Barnesiella* than colitis rats, and they could restore the microbiome to levels similar to those observed in healthy rats [43]. From the view of the curative effect of moxibustion and the whole result of rebalance of intestinal flora, our manuscript has some similarities to this article. However, there are still distinct features or difference in the current research. Firstly, obvious distinction was observed in the fecal microbiota among laboratory mouse, rat, miniature pig, and human. In addition, compared with rat and pig, the homology in mice is the highest [25]. This trend can also be seen in our research (Figure 5(a)). This is very important for metagenomic related research. Additionally, both sequencing data of mice and rats will provide more information for future investigators. Secondly, the treatments in this paper are including not only moxibustion but also electroacupuncture; furthermore we found the treatment of moxibustion can bring more benefits to UC animals. Last but not the least, the acupoints used in the two articles are different. Qin Qi et al. used ST25 and we used ST36 and CV4[43], both studies provide evidence that traditional external therapies can regulate unbalanced gut microbiota, which will help acupuncturist and clinic workers learn more information from the view of traditional Chinese medicine theories. Therefore, both articles would be effectively combined and mutually specified in order to analyze the effect of acupuncture and moxibustion.

### 3.4. The Intestinal Flora Is closely Associated with Treg and Th17 Axis Balance, Indicating That Immune Regulation May Be One of the Most Important Parts within Mechanism during the Interventions of Electroacupuncture and Moxibustion

Treg and Th17 cells, two types of CD4^+^ T cells, which form an immune axis, are closely related to the occurrence and development of UC. Usually, Th17 as T-helper subset is characterized to promote tissue inflammation but Treg is identified to suppress diverse inflammation and immune responses [44]. It is increasingly clear that UC is an autoimmunity disease and regulated by various immune cells in which Treg and Th17 axis is one of the core and central factors. Previous studies concluded that Treg subtype decreased and Th17 subtype increased in UC mice [45]. Treg cells could suppress the inflammatory reactions and against tissue injury and they also can produce some protective cytokines, such as IL-10 and TGF-*β*[46]. While Th17 cells can enhance intestinal autoimmunity progress and lead to tissue destruction [47]. Thus, the unbalance of Treg and Th17 is bound to enhance UC. Acupuncture and moxibustion could restore this unbalance and express well therapeutic effects in UC [24]. The mechanism of acupuncture and moxibustion restoring Treg/Th17 axis is studied in our previously research [45]. In current study, we also found that acupuncture and moxibustion can improve intestinal flora diversity and abundance. Thus, it is interesting to explore if there are any connections between restoration of Treg/Th17 axis and the improvement of intestinal flora diversity. The results showed that the increased intestinal flora diversity and abundance, which contribute obviously to the recovery of UC, were positively correlated with the improvement of Treg cells during electroacupuncture and moxibustion treatment. Meanwhile, Th17, which leads to the occurrence of UC, was suppressed by the two interventions. Additionally, its variations indicated the negative association with alteration of intestinal flora. Thus, our result indicated that immune regulation especially T cells regulation may be one of the most important mechanisms during the interventions of electroacupuncture and moxibustion. In fact, it has been well known that gut microbiome can profoundly affect T cell function and regulate the Treg/Th17 axis. Previous studies showed there are at least three possible ways that the gut microbiome regulates T cells. (1) Symbiotic bacteria can promote T cell differentiating into different subtypes [24, 48]. (2) Gut microbiome can activate or inactivate these T cell [48]. (3) Gut microbiome and their metabolite can affect T cell metabolic pattern which will directly leads to T cell differentiation and activity [44, 48, 49]. In these studies, researchers also proved that microbe-derived short-chain fatty acids induce production of Treg, including butyrate participating in promoting Treg development by facilitating histone H3 acetylation, whereas acetate and propionate facilitate Treg migrating into colon [44]. Considering our results, we suppose that some small metabolites such as SCFAs, which have been proved by our previous study [50, 51], maybe changed after the interventions of electroacupuncture and moxibustion, and which then helps rebalance or restore the Treg/Th17 axis. On the other hand, the Treg and Th17 lymphocytes can affect intestinal flora via regulating inner immunity environment as well [44]. Thus, the crosstalk between T cell and intestinal flora in the gastrointestinal tract will be regulated by acupuncture and moxibustion.

## 4. Conclusion

Our study found that gut microbiome, involving diversity of intestinal flora and the composition and abundance of bacterial communities which are associated with Th17/ Treg cells, could be regulated by electroacupuncture and moxibustion. These findings provide a new approach to understand the relationship between host and microbiome when using some external therapies such as acupuncture and moxibustion.

## 5. Materials and Methods

### 5.1. Ethics Statement

All experimental animals were purchased from the Sichuan Dashuo Experimental Animal Co. Ltd. (license number: SCXK(chuan)2015-030). Committee for Animal experiments of Chengdu University of Traditional Chinese Medicine. All experiments were performed in accordance with the Guide for the Care and Use of Laboratory Animals prepared by the institutional Animal Care and Use Committee of Chengdu University of Traditional Chinese Medicine. All experiments were guided by the Tab of Animal Experimental Ethical Inspection of Laboratory Animal Centre, Chengdu University of Traditional Chinese Medicine. The number of Ethics for this experiment is 2014-07.

### 5.2. Animals and UC Model Establishment

Animals and UC model induced were performed as our previous methods [24, 45]. Briefly, male Kunming mice (23±2 g, 6-8 weeks) were housed in an environmentally controlled vivarium under a 12h light-dark cycle (temperature 23±1°C, humidity 55%-65%). The food and water were cleaned by radiation sterilization. Mice were kept in such environment and allowed to get food and water freely for 1 week. UC was induced by drinking water dissolved 3% Dextran Sodium Sulfate (DSS, 43 kDa, MP Biomedicals) for 7 days. DSS is a classical drug, which will lead to colonic inflammation. The dose of 3% was chosen on the basis of our preliminary experiment which caused highest reproducibility and lowest mortality rate. The 49 mice randomly divided into four groups: control (n=12), UC (n=12), UC with electroacupuncture treatment (n=13), and UC with moxibustion treatment (n=12). One mouse in the electroacupuncture group died probably because of weakness and excessive grabbing and acupuncture stimulation. The animal survival rate is 97.96%.

### 5.3. Electroacupuncture and Moxibustion Treatment

Electroacupuncture and moxibustion treatments were also carried out as our previous study. Treatments were initiated at day 6 after UC model induced. For electroacupuncture, local skin was sterilized by 75% ethanol before treatment. Stainless steel needles (0.25 mm diameter and 13mm length) were inserted into the acupoints “Guanyuan” (CV4) and “Zusanli” (ST36), which were considered to be effective in treating UC according to clinical practices and our previous studies [42, 45, 52]. ST36 was alternately performed at the left or right lower limbs. The locations for these acupoints were determined according to Government Channel and Points Standard GB12346-90 of China and “The Veterinary Acupuncture of China.” The depth of these points was 2mm for CV4 and 3mm for ST36. Needle handles were connected with an electroacupuncture apparatus (Hans-200, China). Stimulating parameters were rarefaction wave, frequency 2/15 Hz, and current strength 0.4-0.6mA. A constant electrical stimulus was applied 15min once a day for consecutive 5 days. For moxibustion, a small moxa cone (diameter 0.4cm) for animals was placed on the moxa strip fixing device made by ourselves [24]. The distance between the skin and moxa cone was about 1.2-1.5 cm in which mild-warm moxibustion (38±1°C) was operated and skin was not burned. Each acupoint was treated for 10 min every day. A constant moxibustion was applied 15min once a day for consecutive 5 days. Mice in the control and UC groups did not get any treatments, but they were sham-handled in the same way and time as mice in the acupuncture and moxibustion groups.

### 5.4. General Assessment of Colitis

Severities of colitis and therapeutic effects were assessed by general manifestation, occult blood, and disease activity index (DAI). General manifestations include weight, complexion, psychomotility, and fecal appearance. Occult blood was tested by benzidine and assessments were graded into 4 degrees: strong positive (+++), dark blue is expressed in 1min; positive (++), blue or bluish-green is expressed after 1min; slight positive (+) is expressed after 5min; and negative (-), no color. Programs of DAI include weight ratio, stool consistency, and occult blood.

DAI score was graded as follows: 0, normal in 3 programs; 1, only weight ratio decrease 1-5%; 2, weight ratio decrease 6-10%, soft excrement, and occult blood; 3, weight ratio decrease 11-15%, soft excrement, and occult blood; 4, greater than or equal to 16% of ratio, loose excrement, and bloody stools.

### 5.5. Tissue Collection

All mice were sacrificed on the 9^th^ day after the final treatment (that means all the treatments last for 5 days). The abdomen of mice was sterilized by iodophor and 75% alcohol after barbering in super clean bench. Then whole colon from the anus to the cecum proximal end was removed and cut open along the lengthwise. The stools in colon were collected into sterile tubes and immediately frozen in liquid nitrogen for detection of diversity of intestinal flora. The colonic tissues were washed by 0.9% saline and distal colon segment which is 2-3cm upward from the anus was cut and fixed in 4% paraformaldehyde and then dehydrated by gradient alcohol and paraffin-embedded.

### 5.6. Histopathological Examination of Mice Colon

Paraffin-embedded colon was cut into sections (thickness 3-5*μ*m). The sections were deparaffinized by xylene and the xylene was removed by alcohol. Then sections were stained with haematoxylin and eosin (HE) and observed under an optical microscope (Mike Audi BA200Digital).

### 5.7. Microbial Diversity Analysis

#### 5.7.1. DNA Extraction and PCR Amplification

Microbial DNA was extracted from stool samples using the OMEGA-soil DNA Kit (Omega Bio-tek, Norcross, GA, U.S.) according to manufacturer's protocols. The V4-V5 region of the bacteria 16S ribosomal RNA gene was amplified by PCR (95°C for 2 min, followed by 25 cycles at 95°C for 30 s, 55°C for 30 s, and 72°C for 30 s, and a final extension at 72°C for 5 min) using primers 338F 5′-ACTCCTACG-GGAGGCAGCAG-3′ and 806R 5′-GGACTACHVGGGTWTCTAAT-3′. PCR reactions were performed in triplicate 20 *μ*L mixtures containing 4 *μ*L of 5 × FastPfu Buffer, 2 *μ*L of 2.5 mM dNTPs, 0.8 *μ*L of each primer (5 *μ*M), 0.4 *μ*L of FastPfu Polymerase, and 10 ng of template DNA.

#### 5.7.2. Illumina MiSeq Sequencing

Amplicons were extracted from 2% agarose gels and purified using the AxyPrep DNA Gel Extraction Kit (Axygen Biosciences, Union City, CA, U.S.) according to the manufacturer's instructions and quantified using QuantiFluor™-ST (Promega, U.S.). Purified amplicons were pooled in equimolar and paired-end sequences (2 × 250) on an Illumina MiSeq platform according to the standard protocols. Raw reads were deposited into the NCBI Sequence Read Archive (SRA) database (Accession Number: SRP126121:PRJINA420628).

#### 5.7.3. Processing of Sequencing Data

Raw fastq files were demultiplexed and quality-filtered using QIIME (version 1.17) with the following criteria: (i) 300 bp reads were truncated at any site receiving an average quality score <20 over a 50 bp sliding window, discarding the truncated reads that were shorter than 50bp. (ii) Exact barcode matching, 2 nucleotide mismatch in primer matching, and reads containing ambiguous characters were removed. (iii) Only sequences that overlap longer than 10 bp were assembled according to their overlap sequences. Reads which could not be assembled were discarded. Operational Units (OTUs) were clustered with 97% similarity cutoff using UPARSE (version 7.1 http://drive5.com/uparse/) and chimeric sequences were identified and removed using UCHIME. The taxonomy of each 16S rRNA gene sequence was analyzed by RDP Classifier (http://rdp.cme.msu.edu/) against the silva (SSU115) 16S rRNA database using confidence threshold of 70%[53].

#### 5.7.4. Correlation Analysis

We count the correlation between the intestinal flora diversity and Treg or Th17 cells. The data of intestinal flora include Sobs, Ace, Chao, and Shannon. The number of Treg and Th17 positive cells was tested by flow cytometry which have been reported in our previous reports [45]. These data were tested by correlation analysis in SPSS 20.0.

## Figures and Tables

**Table 1:** UC disease activity index.

Body weight loss(%)	Characteristics of feces	Fecal occult blood/gross fecal blood	Scoring
0	Normal	Normal	0
1-5			1
6-10	Loose Fecal	Fecal occult blood	2
11-15			3
>16	Watery feces	Naked eye bloody feces	4

**Table 2:** Comparison of DAI in each group (Means±SD).

Group	N	After modeling	After treatment
Control	12	—	—
Model	12	3.77±1.24	3.62±1.26
Electro-acupuncture	12	3.75±1.36	1.75±1.22^△△^
Moxibustion	12	3.62±1.12	1.77±1.17^△△^

Note: ^△△^compare with model group (P<0.01).

**Table 3:** Community composition and proportion of intestinal flora in each group on genus level (%).

	Control	Model	Electro-acupuncture	Moxibustion
*norank_f_Bacteroidales_S24-7_group*	23.35	15.37	7.01	14.18
*Lachnospiraceae_NK4A136_group*	9.56	12.45	21.31	16.74
*Lactobacillus*	19.61	2.25	8.61	12.35
*Bacteroides*	1.44	11.22	15.96	10.99
*unclassified_f_Lachnospiraceae*	4.09	6.05	14.36	12.37
*uncultured_f_Lachnospiraceae*	5.91	5.37	7.01	6.92
*Odoribacter*	0.91	6.23	3.23	2.20
*Allobaculum*	0.00	10.00	0.00	1.20
*Helicobacter*	1.64	1.43	2.34	3.86
*Lachnoclostridium*	1.02	2.34	2.06	2.97
*uncultured_f_Ruminococcaceae*	1.70	2.06	2.26	1.46
*Streptococcus*	0.31	5.25	0.17	0.09
*Lachnospiraceae_UCG-001*	4.20	0.00	0.00	0.00
*Oscillibacter*	1.13	0.83	1.42	1.01
*Mucispirillum*	1.12	1.57	1.03	0.61
*others*	23.98	17.59	13.25	13.13

## Data Availability

The data used to support the findings of this study are available from the corresponding author upon request.

## Supplementary Materials

Supplementary Materials Flow cytometry results of the percentage of Treg cells (CD4^+^CD25^+^FOXP3^+^) and Th17 (CD3^+^CD8^+^IL-17^+^) cells among CD4^+^ T cells in different groups were shown in the supplementary material. Briefly, lymphocytes from spleen of each group were stained with labeled anti-mice antibodies. For Treg analysis, FITC (BD Biosciences, USA), PE (BD Biosciences, USA), and Foxp3 PerCP-cy5.5 (eBioscience, USA) were used for dying CD4+CD25+Foxp3+Treg cells and their corresponding isotype controls. For Th17 analysis, lymphocytes from spleen of each group were stimulated with ionomycin, PMA, and monensin for 5 h. FITC, PE, IL-17A PerCP-cy5.5 (eBioscience, USA) were used for dying CD3+CD8+IL-17+Th17 cells and their respective isotype controls (from BD Biosciences, USA and eBioscience, USA respectively). The flow cytometry is FC-500(Beck Coulter, USA). The percentage of Treg (CD4+CD25+Foxp3+Treg /CD4+ T cells) in model group decreased (P<0.05). Treg cells increased after acupuncture or moxibustion treatment (P<0.05). On the contrary, the percentage of Th17 cells significantly increased (P<0.05) in UC model mice while decreasing by both acupuncture and moxibustion (P<0.05).
