# Supplementary material for: Gut Microbiota: A New Strategy to Study the Mechanism of Electroacupuncture and Moxibustion in Treating Ulcerative Colitis
Source: Evid Based Complement Alternat Med. 2019 Jul 1;2019:9730176. doi: 10.1155/2019/9730176 (PMC6632505; doi:10.1155/2019/9730176)
Supplement: Supplementary Materials — Flow cytometry results of the percentage of Treg cells (CD4+CD25+FOXP3+) and Th17 (CD3+CD8+IL-17+) cells among CD4+ T cells in different groups were shown in the supplementary material. Briefly, lymphocytes from spleen of each group were stained with labeled anti-mice antibodies. For Treg analysis, FITC (BD Biosciences, USA), PE (BD Biosciences, USA), and Foxp3 PerCP-cy5.5 (eBioscience, USA) were used for dying CD4+CD25+Foxp3+Treg cells and their corresponding isotype controls. For Th17 analysis, lymphocytes from spleen of each group were stimulated with ionomycin, PMA, and monensin for 5 h. FITC, PE, IL-17A PerCP-cy5.5 (eBioscience, USA) were used for dying CD3+CD8+IL-17+Th17 cells and their respective isotype controls (from BD Biosciences, USA and eBioscience, USA respectively). The flow cytometry is FC-500(Beck Coulter, USA). The percentage of Treg (CD4+CD25+Foxp3+Treg /CD4+ T cells) in model group decreased (P<0.05). Treg cells increased after acupuncture or moxibustion treatment (P<0.05). On the contrary, the percentage of Th17 cells significantly increased (P<0.05) in UC model mice while decreasing by both acupuncture and moxibustion (P<0.05). [file 9730176.f1.pptx]

## Slide 1
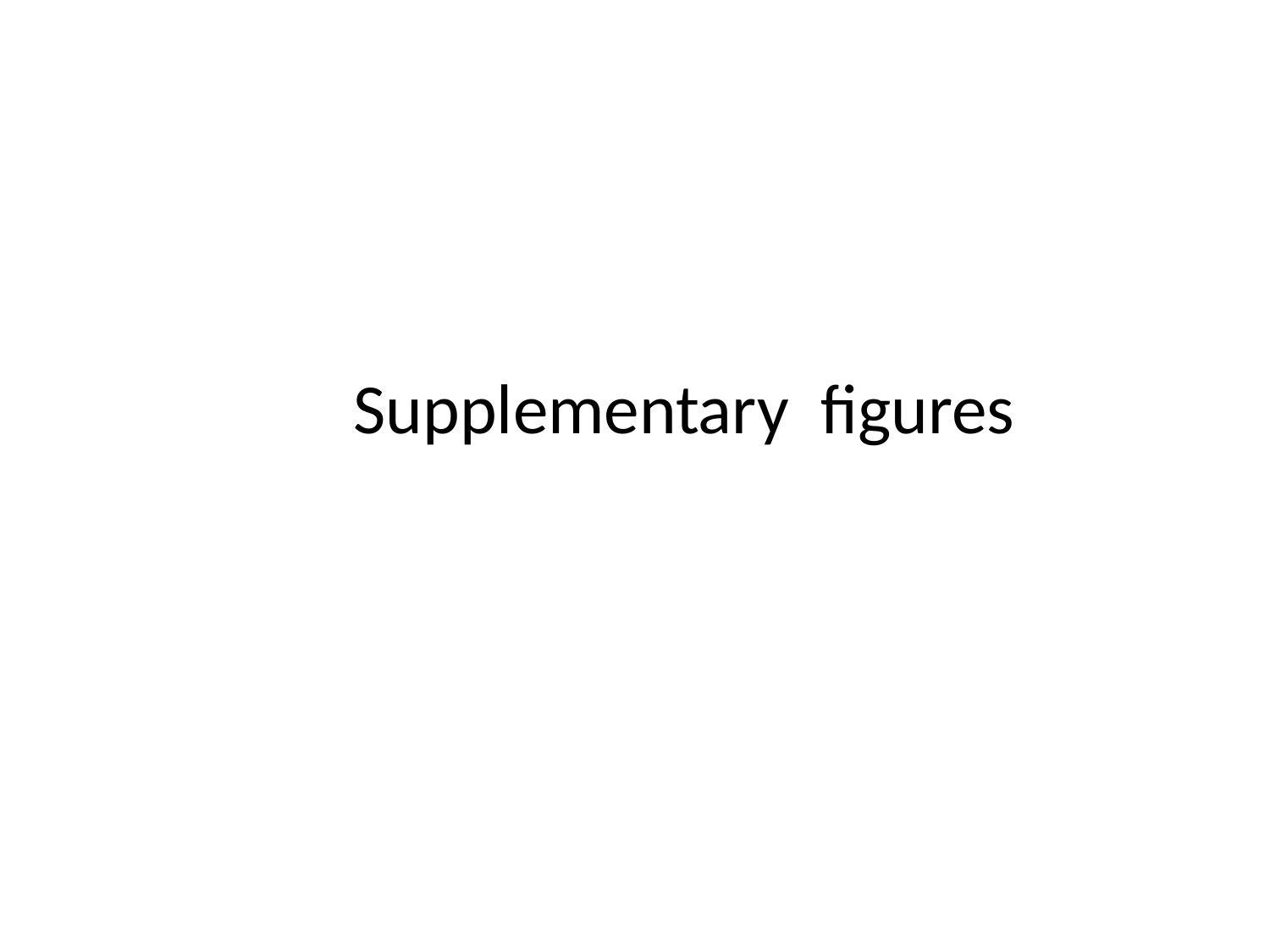

#
Supplementary figures

## Slide 2
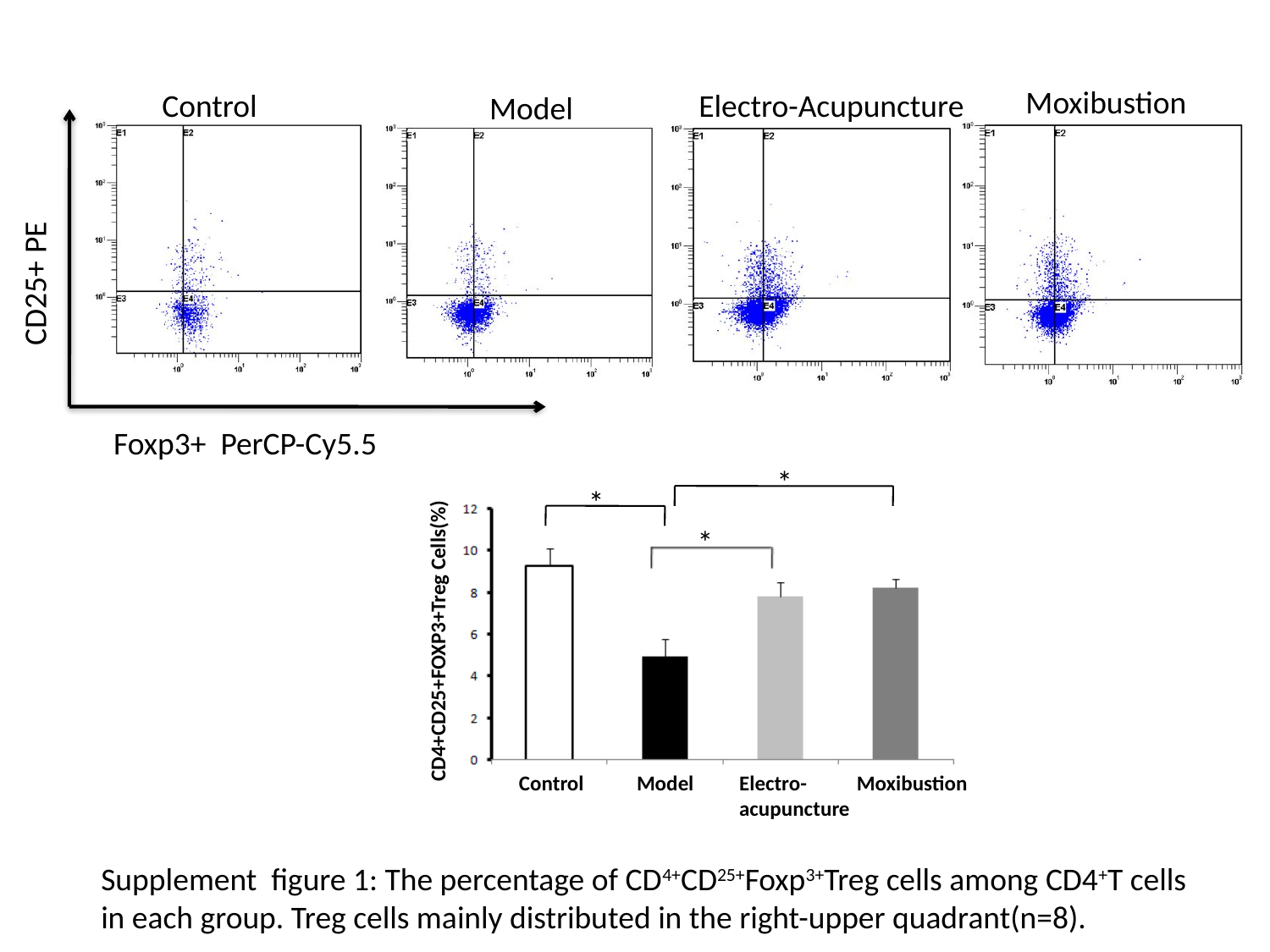

Moxibustion
Control
Electro-Acupuncture
Model
Foxp3+ PerCP-Cy5.5
CD25+ PE
*
*
Control
Model
Electro-
acupuncture
Moxibustion
*
CD4+CD25+FOXP3+Treg Cells(%)
Supplement figure 1: The percentage of CD4+CD25+Foxp3+Treg cells among CD4+T cells
in each group. Treg cells mainly distributed in the right-upper quadrant(n=8).

## Slide 3
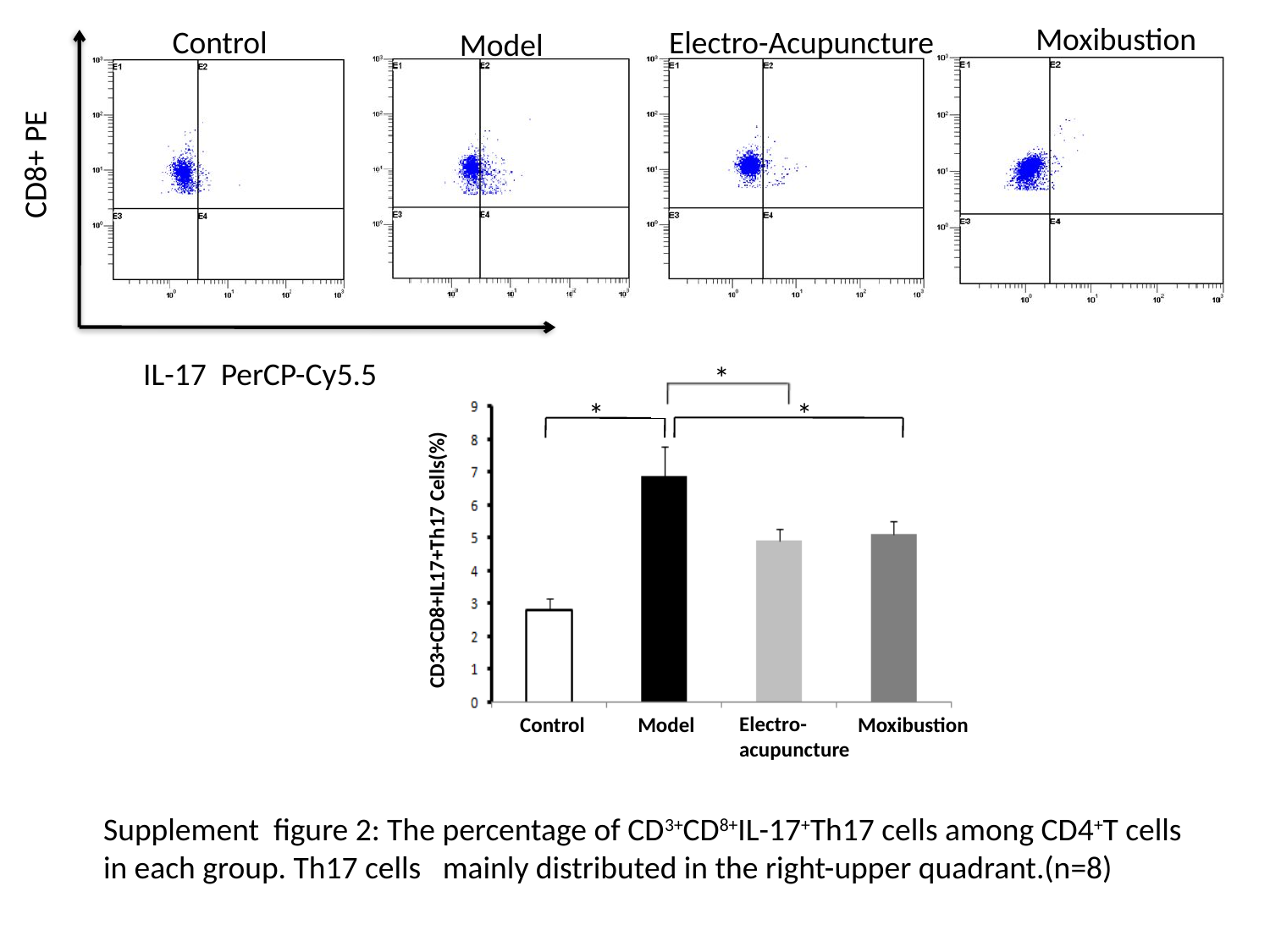

Moxibustion
Control
Electro-Acupuncture
Model
CD8+ PE
IL-17 PerCP-Cy5.5
*
*
*
CD3+CD8+IL17+Th17 Cells(%)
Control
Model
Moxibustion
Electro-
acupuncture
Supplement figure 2: The percentage of CD3+CD8+IL-17+Th17 cells among CD4+T cells
in each group. Th17 cells mainly distributed in the right-upper quadrant.(n=8)
